# Effects of positive end-expiratory pressure on lung ventilation/ perfusion matching: a clinical study

**DOI:** 10.1186/cc14336

**Published:** 2015-03-16

**Authors:** N Eronia, T Mauri, G Bellani, R Marcolin, S Marocco Arrigoni, G Grasselli, A Pesenti

**Affiliations:** 1 San Gerardo Hospital, Monza, Italy; 2 IRCC Ca' Granda Maggiore Policlinico Hospital, Milan, Italy

## Introduction

Positive end-expiratory pressure (PEEP) exerts multiple protective effects in hypoxemic critically ill patients: PEEP can increase end-expiratory lung volume (EELV) and induce recruitment, thus reducing lung strain and opening and closing of alveoli and potentially improving the ventilation/perfusion matching. In particular, estimation of PEEP-induced ventilation/perfusion matching might help identify the optimal PEEP setting, but bedside non-invasive methods are few and complex to be applied in daily clinical practice. Electrical impedance tomography (EIT) is a non-invasive bedside technique that claims to track global and regional changes in perfusion and ventilation over time. In the present study we aimed at verifying the effects of PEEP on ventilation/perfusion matching, as assessed by EIT, in acute respiratory failure patients.

## Methods

We enrolled 20 intubated critically ill patients undergoing controlled mechanical ventilation, sedated, paralyzed and with PaO_2_/FiO_2_ ≤300 at PEEP ≥5 cmH_2_O. We started EIT monitoring (Pulmovista500®; Dräger Medical GmbH, Lübeck, Germany) and applied two PEEP levels (clinical and clinical + 5 cmH_2_O) for 20 minutes each. We collected ventilatory and EIT parameters and, by offline analysis, we calculated the increase of EELV at higher PEEP and the EIT-based indexes of ventilation heterogeneity (VtHetend-insp) and of the regional homogeneity of ventilation/perfusion matching (HA/P).

## Results

Patients were 62 ± 12 years old, mean PaO_2_/FiO_2_ was 197 ± 52, lower PEEP level was 7 (7 to 9) cmH_2_O, while higher PEEP was 12 (12 to 14) cmH_2_O (*P *< 0.001). At higher PEEP, EELV increased (391 (334 to 555) ml vs. PEEP low, considered as baseline, *P *< 0.001); VtHetendinsp was reduced (1.8 (1.5 to 2.4) vs. 2.2 (1.8 to 2.6), *P *< 0.001) and HA/P increased (0.29 ± 0.19 vs. 0.2 ± 0.15, *P *< 0.05). Interestingly, the increase of HA/P was significantly correlated with the decrease of VtHetendinsp (*r *= -0.48, *P *< 0.05). Moreover, patients with higher potential for improvement of ventilation/perfusion matching (that is, patients with increase of HA/P >16%) had higher baseline VtHetend-insp (2.6 (2.3 to 4.8) vs. 1.9 (1.5 to 2.1), *P *< 0.01) and lower compliance of dependent lung regions (Crsdep, 13 ± 3 ml/cmH_2_O vs. 18 ± 6 ml/cmH_2_O, *P *< 0.05), as compared with patients with smaller improvement.

## Conclusion

EIT might represent a feasible, bedside method to estimate PEEP-induced improvement in ventilation/perfusion matching. Assessing regional ventilation and mechanical lung properties might help identify patients who would benefit more from higher PEEP.

